# Derivation of Two New Human Embryonic Stem Cell Lines from Nonviable Human Embryos

**DOI:** 10.4061/2011/765378

**Published:** 2011-05-22

**Authors:** Svetlana Gavrilov, Darja Marolt, Nataki C. Douglas, Robert W. Prosser, Imran Khalid, Mark V. Sauer, Donald W. Landry, Gordana Vunjak-Novakovic, Virginia E. Papaioannou

**Affiliations:** ^1^Department of Genetics and Development, College of Physicians and Surgeons of Columbia University, 701 West 168th Street, New York, NY 10032, USA; ^2^Department of Biomedical Engineering, College of Physicians and Surgeons of Columbia University, New York, NY 10032, USA; ^3^Department of Obstetrics and Gynecology, College of Physicians and Surgeons of Columbia University, New York, NY 10032, USA; ^4^Division of Experimental Therapeutics, Department of Medicine, College of Physicians and Surgeons of Columbia University, New York, NY 10032, USA

## Abstract

We report the derivation and characterization of two new human embryonic stem cells (hESC) lines (CU1 and CU2) from embryos with an irreversible loss of integrated organismic function. In addition, we analyzed retrospective data of morphological progression from embryonic day (ED) 5 to ED6 for 2480 embryos not suitable for clinical use to assess grading criteria indicative of loss of viability on ED5. Our analysis indicated that a large proportion of *in vitro* fertilization (IVF) embryos not suitable for clinical use could be used for hESC derivation. Based on these combined findings, we propose that criteria commonly used in IVF clinics to determine optimal embryos for uterine transfer can be employed to predict the potential for hESC derivation from poor quality embryos without the destruction of vital human embryos.

## 1. Introduction

Human embryonic stem cells (hESCs) are the ultimate model for *in vitro* study of cell growth and differentiation and the most promising candidates for cell therapy in regenerative medicine due to their high proliferative capacity and ability to differentiate into lineages of all three embryonic germ layers [1]. hESCs are rapidly emerging as valuable screening platforms for drug toxicity and tools for drug discovery [2–4]. 

Recent advances in generation of induced pluripotent stem (iPS) cells [5–8] have shifted focus from hESC to iPS cells as more likely candidates for clinical use, as they have the potential to generate patient-specific therapy and avoid the unwanted immune response in the host [5]. However, in a recent study the question of developmental equivalence between hESC and human iPS cells was raised. Using gene expression profiling, it was demonstrated that despite the fact that hESC and human iPS cells share pluripotency markers, their expression signatures are distinct [9]. It is unresolved whether the small percentage of genes that is differentially expressed could result in a biologically significant difference [5, 10]. Importantly, two recent studies demonstrated that iPS cells retain an epigenetic memory relating to their cells of origin that restricts their differentiation potential [11, 12]. While iPS cell technology does seem a very promising avenue for bringing stem cell therapy to the bedside, it is important to continue studying hESC, because it is difficult to predict whether hESC or iPS cells will be more effective for therapeutic application. 

hESC lines are conventionally derived from the inner cell mass (ICM) of viable human preimplantation embryos. Our group has proposed one of several strategies for derivation of hESC without the destruction of viable embryos [13]. During routine *in vitro* fertilization (IVF) procedures, a large proportion of embryos fail to develop properly [14–16] and are discarded because they are unsuitable for embryo transfer [17, 18]. We and others have found that many of these embryos have suffered an irreversible loss of integrated organismic function and have defined them on that basis as nonviable or organismically dead [19, 20]. In our study, such ED6 embryos did not progress upon extended *in vitro *culture although more than 80% of them contained living cells as assessed by the use of vital dyes [20]. Thus, we have proposed that hESC lines could be derived from these nonviable embryos and have suggested that ethical guidelines for essential organ donation could be employed for the clinical application of this paradigm [13, 20–22]. To date, two groups have successfully derived hESC from poor quality embryos that were nonviable by our criteria. In the first report, a single, stable hESC line was derived from 132 arrested embryos [23], and in the second, eleven lines were derived from 413 poor quality embryos rejected for clinical use [24]. All 12 hESC lines were karyotypically normal and pluripotency and their differentiation potential was demonstrated both *in vitro* and *in vivo*.

Our objectives were first to extend this work by deriving new hESC lines from discarded, nonviable embryos at ED6 and secondly to define morphological criteria for nonviability a day earlier, on ED5, in order to allow the earlier derivation of hESC when a greater number of viable cells are likely to be present. Here, we describe two new hESC lines derived from nonviable ED6 embryos and in a retrospective study identify a subgroup of nonviable, cavitated embryos with the potential to yield hESC lines. By targeting derivation efforts on this subgroup, derivation success rate might be increased.

## 2. Materials and Methods

### 2.1. Feeder Cells and Culture Media

Commercial irradiated CF1 mouse embryo fibroblasts (MEFs)(Global Stem Cell, Rockville, MD) were plated on 0.1% gelatin (Sigma Aldrich, St Louis, MO) coated 4-well dishes (NUNC, Rochester, NY) at 150,000 cells per well. MEFs were cultured in MEF media consisting of DMEM (Invitrogen, Carlsbad, CA), 10% FBS (Hyclone, Logan, UT), 100 mM L-glutamine (Invitrogen), and 1% penicillin/streptomycin (Invitrogen). 

hESC derivation media consisted of Knockout DMEM (Invitrogen), 17% Knockout Serum Replacement (KSR, Invitrogen) with 3% FBS (Hyclone), 10 ng/mL bFGF (Invitrogen), 100 *μ*M beta-mercaptoethanol (Invitrogen), 100 mM nonessential amino acids (Invitrogen), 1 mM L-glutamine (Invitrogen), and 1% penicillin/streptomycin (Invitrogen). hESCs were cultured in the same media with the exception of 20% KSR, 20 ng/mL bFGF, and no FBS. Quinn's HEPES medium (Q-HEPES) and Quinn's Advantage Protein Plus Blastocyst Medium (QBlast) (both from Sage Media, Cooper Surgical, Pasadena, CA) were used for embryo recovery or washes and short-term culture, respectively.

### 2.2. Embryos

Approvals were obtained from the Institutional Review Board (IRB) and the Embryonic Research Committee at Columbia University to collect nonviable embryos. The embryos were generated by IVF for the purpose of treating infertile couples with assisted reproduction at the Center for Women's Reproductive Care (CWRC). For all embryos used in this study, patients' written consent was obtained prior to the IVF procedure and patients' records were deidentified. Patients agreed to allow research to be performed upon any and all nonviable embryos destined to be discarded. In total, 375 embryos from 87 patients were declared nonviable and were available for this study. Only embryos meeting criteria of nonviability were subject to attempted hESC derivation.

All patients underwent routine controlled ovarian hyperstimulation with human gonadotropins to effect follicle growth and development. Oocyte retrieval, fertilization, and assessment of embryo development were performed by CWRC as previously described [20]. On embryonic day 6 (ED6), embryos that had arrested or failed to divide normally were rejected for clinical use by the CWRC laboratory. They were transported to the laboratory as previously described [20] and used for hESC derivation.

### 2.3. Embryo Plating and Outgrowth Culture

Embryos were recovered as previously described [20] and placed in 20 *μ*L drop cultures with QBlast. Assessment was performed under phase and brightfield optics to determine the morphological categories [20] and to triage embryos for derivation. Derivation was attempted only for 159 embryos that showed signs of compaction and/or cavitation. These embryos were treated with acid Tyrode's (AT) (Sigma) solution to remove the zona pellucida (ZP), washed briefly in Q-HEPES and plated onto dishes of inactivated MEFs in hESC derivation media, and incubated at 37°C in 5% CO_2_ in air. Media was changed every second day. Outgrowths displaying hESC-like morphology were observed 2–14 days postplating. When hESC lines were successfully established, hESC derivation media was exchanged for hESC culture media to prevent spontaneous differentiation. Initial passaging of outgrowths and subsequent established hESC lines was carried out by microdissection (“cut and paste” subculture). Typically, microdissection was performed every 5–7 days; cystic and differentiated material was removed and undifferentiated areas of colonies were cut into several pieces, collected by aspiration and transferred onto fresh feeders.

### 2.4. Immunofluorescence

hESC colonies and adhered embryoid bodies were fixed in Formalin-Free Fixative (Sigma) or 3.7% formaldehyde in PBS for 20 minutes, permeabilized with 0.1% Triton in PBS for 10 minutes for intracellular stains and blocked with 10% goat/horse serum for 45 minutes. Cells were incubated with primary antibody at room temperature for 1 hour, washed, and incubated with an appropriate secondary antibody for 30 minutes. After washing, the nuclei were stained with 4,6-diamidino-2-phenylindole dilactate (DAPI, Invitrogen) for 2 minutes, and the cultures were observed on a fluorescent microscope (IX81, Olympus, Center Valley, PA). Oct3/4 antibody, neuron-specific beta-III tubulin (TuJ-1) antibody and goat IgG secondary antibody were from R&D Systems (Minneapolis, MN). Stage-specific embryonic antigen-3 (SSEA-3), SSEA-4, tumor rejection antigen 1–60 (TRA 1–60), TRA 1–81 antibodies, and mouse IgG/IgM secondary antibody were from Chemicon/Millipore (Billerica, MA). Alpha-1-fetoprotein (AFP) antibody was from DakoCytomation (Glostrup, Denmark), and smooth muscle actin (SMA) antibody was from Invitrogen.

### 2.5. Quantitative Real-Time Reverse-Transcription Polymerase Chain Reaction (qRT-PCR)

Total RNA was extracted from hESC and embryoid bodies using RNeasy mini-kits (Qiagen, Valencia, CA), followed by treatment with DNase (Invitrogen). Total RNA was reverse-transcribed into cDNA with random hexamers using SuperScript III First-Strand Synthesis System (Invitrogen). Expression of *POU5F1/OCT4*, *SOX2*, *NANOG*, *BRACHYURY*, *AFP* and *PAX6* was quantified using the ABI Prism 7900 real-time system (Applied Biosystems, Foster City, CA) and normalized to the expression of housekeeping gene glyceraldehyde-3-phosphate-dehydrogenase (*GAPDH*). Standard cycling conditions were used for all assays (TaqMan chemistry, Applied Biosystems). Primer sequences for the human *NANOG* gene [25] were 5′-TGAGCTGGTTGCCTCATGTTAT-3′ (forward primer), 5′-GAAGGAAAAGTATCAAGAAATTGGGATA-3′ (reverse primer), 5′-ATGCAGGCAACTCA-3′ (FAM/non-fluorescent quencher labeled MGB probe) (Applied Biosystems). Primers and probes for *POU5F1/OCT4* (Hs00742896_s1), *SOX2* (Hs00602736_s1), *T/BRACHYURY* (Hs00610080_m1), *AFP* (Hs00173490_m1), *PAX6* (Hs00240871_m1), and *GAPDH* (4310884E, VIC/TAMRA labeled) were purchased from Applied Biosystems.

### 2.6. *In Vitro* Differentiation Assay

Near confluent hESCs were microdissected as described, transferred to nonadherent plates and allowed to differentiate spontaneously by embryoid body formation in Knockout DMEM (Invitrogen), supplemented with 20% FBS (Hyclone), 100 *μ*M beta-mercaptoethanol (Invitrogen), 100 mM nonessential amino acids (Invitrogen), 1 mM L-glutamine (Invitrogen), and 1% penicillin/streptomycin (Invitrogen). Embryoid bodies were cultured up to 5 weeks with media change twice per week. For gene expression analyses, embryoid bodies were harvested on days 4, 8, 14, and 21. For immunofluorescence, embryoid bodies were transferred to gelatin-coated glass-bottom culture dishes (MatTek, Ashland, MA) after 3 weeks and cultured for an additional week to achieve differentiated cell outgrowth. For histology, embryoid bodies were harvested after 5 weeks, washed with PBS, fixed in 3.7% formaldehyde in PBS, and embedded in paraffin. Ten *μ*m-thick sections were stained with hematoxylin-eosin (HE) or Masson's trichrome.

### 2.7. *In Vivo* Differentiation Assay

For the generation of teratomas, contents of two wells of a subconfluent 6-well dish (approximately 10^5^-10^6^ cells) were subcutaneously injected per mouse into the neck region of 4–6 weeks old SCID beige mice (Harlan, Indianapolis, IN) (*n* = 4 per experimental group). Animals were palpated weekly for development of tumors. 12 to 14 weeks postinjection, suspected tumors were removed, fixed in 4% paraformaldehyde or Bouin's fixative solution and embedded in paraffin. Ten *μ*m-thick sections were stained with HE, Masson's trichrome, or processed for immunohistochemistry. All experiments involving laboratory animals were performed under protocols approved by the Columbia University Institutional Animal Care and Use Committee.

### 2.8. Karyotyping

Standard G-band karyotyping was performed by Columbia University Cytogenetics Laboratory.

### 2.9. Retrospective ED5/ED6 Data

With IRB approval, de-identified records of human embryos generated for IVF were examined. Morphological progression from ED5 to ED6 was analyzed retrospectively in embryos that were not suitable for clinical use on ED5 and were reevaluated on ED6. Embryos transferred or cryopreserved on ED5 were also included in the analysis. Most of the embryos analyzed were in group culture, thus morphological categories from ED5 and ED6 were compared as cohort populations. Morphological categories used in the CWRC for clinical grading on ED5 and ED6 were deg = degenerated (an embryo showing pronounced signs of degeneration), frag = fragmented, 1C = single-celled embryo (1C), MC = multicell (embryo containing 2 or more blastomeres with or without cellular fragmentation), comp = embryo showing some sign of compaction, EB = embryo with a small cavity, the first sign of blastocyst formation, B = embryo with a large cavity and HB = hatching blastocyst, embryo with a large cavity and at least one cell protruding through the ZP. All embryos in categories B and HB, which represent blastocyst formation, were given overall grades of good, fair, or poor and in addition, the quality of inner cell mass (ICM) and trophectoderm (TE) was graded from A to D. This is in accordance with the American Society for Reproductive Medicine/Section for Assisted Reproduction Technologies blastocyst grading system, per standard CWRC practice.

## 3. Results

### 3.1. Derivation and Characterization of New hESC Lines

**Figure 1:**
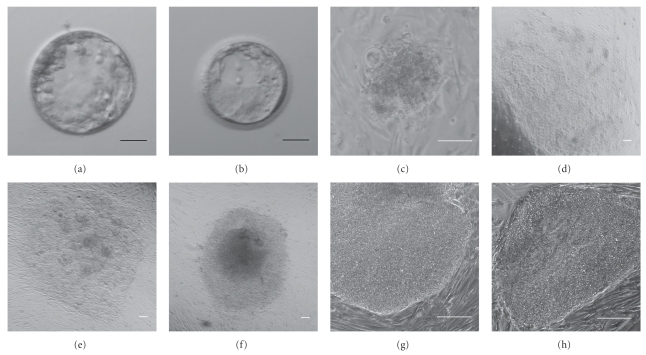
Nonviable ED6 embryos, showing cavitation but either no or highly abnormal ICMs, initial outgrowths, and typical colony appearance. Brightfield images of embryos used for CU1 (a) and CU2 (b) hESC derivation. Phase contrast images of representative initial outgrowths two (c), five (d), or 14 days (e) postplating and typical established hESC colonies (f, g, h). Scale bar = 20 *μ*m (a, b); 100 *μ*m (c–h).

In our previous study, we categorized nonviable ED6 embryos on the basis of gross morphology and determined the number of vital and nonvital cells contained in each embryo [20]. Although morphological categorization was generally of limited value in predicting cell number, a higher number of living cells was associated with cavitation, suggesting that cavitation might predict greater potential for success of hESC derivation. This is in line with a previous study where derivation efficiency was lower for cleavage-arrested embryos than for embryos arrested following cavitation [26]. In preliminary experiments (data not shown), we observed little or no attachment when early-arrested nonviable embryos were plated on MEFs. Thus nonviable ED6 embryos were triaged using morphological categories as previously described [20], and only embryos showing signs of compaction and/or cavitation with no or an abnormal ICM (159/375) were plated on MEFs to attempt hESC derivation. Initial embryo outgrowths were observed from 2 to 14 days after plating on MEFs. In total, 83 embryos gave rise to primary colonies (p0); of these 69 outgrowths deteriorated prior to first passaging, 14 survived the first passage, three survived the second passage, and two survived the third passage and beyond, resulting in the hESC lines CU1 and CU2 (Figure 1). Both CU1 and CU2 hESC colonies display typical stem-cell-like morphology. Karyotype analysis revealed a normal female karyotype for CU1 at passage 9. CU2 showed a normal female karyotype at passage 8 (46, XX) with a low level of mosaicism for chromosome 18 monosomy (3/31 cells; Supplementary Figure  S1 available online at doi: 10.4061/2011/765378). Self-renewal capacity was demonstrated by successfully propagating both hESC lines for more than 30 passages.

**Figure 2:**
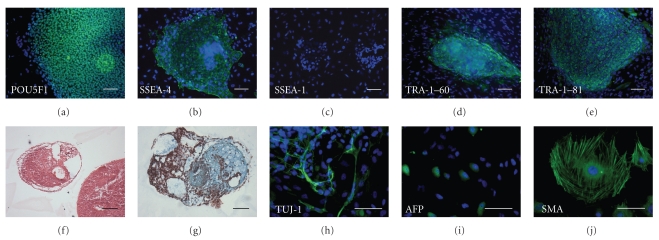
Characterization of CU1 hESC line. Immunohistochemical analysis with DAPI counterstain revealed that hESC colonies expressed pluripotency markers POU5F1/OCT4 (a), SSEA-4 (b), TRA1-60 (d), TRA-1-81 (e) (green fluorescence) and were negative for differentiation marker SSEA-1 (c). hESC cultured as embryoid bodies differentiated into all three germ layers. Hematoxylin and eosin (f), Masson's trichrome (g), immunohistochemistry for neuron-specific beta-III tubulin (TUJ-1) (h), alpha fetoprotein (AFP) (i), and smooth muscle actin (SMA) (j) Scale bar = 100 *μ*m.

**Figure 3:**
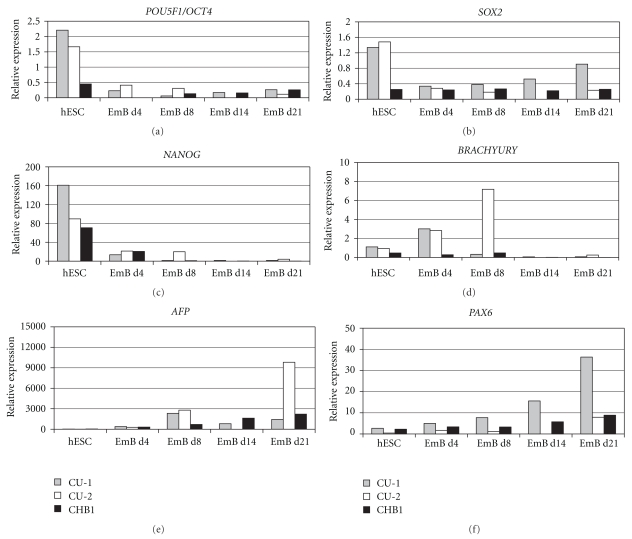
Gene expression analysis by qRT-PCR. Expression of pluripotency markers *POU5F1/OCT4 *(a), *SOX2* (b), *NANOG *(c) and differentiation markers *BRACHYURY* (e), *AFP* (f), and *PAX6* (g) in undifferentiated CU1, CU2, and CHB1 hESC colonies (hESC) and corresponding embryoid bodies (EmB) harvested on days 4, 8, 14, and 21 (EmBd4, EmBd8, EmBd14, and EmBd21, resp.). Expression levels were normalized to expression of *GAPDH*.

Characterization of CU1 and CU2 hESC lines demonstrated the presence of specific hESC markers of the undifferentiated state [25] such as transcription factor POU5F1/OCT4, keratan surface antigens TRA-1–60 and TRA-1–81, glycolipid antigen SSEA-4, and the absence of glycolipid antigen SSEA1, commonly expressed upon hESC differentiation (Figure 2 and Supplementary Figure  2). Similar expression of cell surface antigens was noted for the previously characterized CHB1 line, established from a cleavage-arrested poor quality embryo [26]. Quantitative real-time RT-PCR was used to assess the expression of genes in undifferentiated CU1 and CU2 hESC lines and in differentiated embryoid bodies derived from them. The CHB1 line was used as a control for comparison of gene expression levels in undifferentiated hESC and in differentiated embryoid bodies (Figure 3). Quantitative real-time RT-PCR analysis demonstrated similar or higher levels of expression of pluripotency genes *POU5F1*/*OCT4*, *NANOG*, and *SOX2* in undifferentiated CU1 and CU2 cells when compared with undifferentiated CHB1 line (Figure 3). Upon differentiation into embryoid bodies, the expression of *POU5F1*/*OCT4*, *NANOG*, and *SOX2* was downregulated while the markers of differentiation *BRACHYURY* (mesodermal lineage); alpha fetoprotein (*AFP*; endodermal lineage), and *PAX6* (ectodermal lineage) were upregulated in both CU1 and CU2 hESC lines (Figure 3). Similarly, differentiated embryoid bodies expressed neuron-specific beta-III tubulin (TUJ-1), AFP, and smooth muscle actin (SMA) as assessed by immunofluorescence (Figure 2 and Supplementary Figure  2). Both CU1 and CU2 lines differentiated into derivatives of all three embryonic germ layers *in vitro*. CU1 and CU2 hESC were injected into SCID beige mice (*n* = 4 per experimental group) and no palpable tumors were detected from 12 to 14 weeks postinjection. The analyses of suspected tumors revealed the presence of hibernoma (3 out of 8) at the site of injection while no teratomas were identified upon histological examination (data not shown).

### 3.2. Retrospective Analysis of Embryo Progression

**Figure 4:**
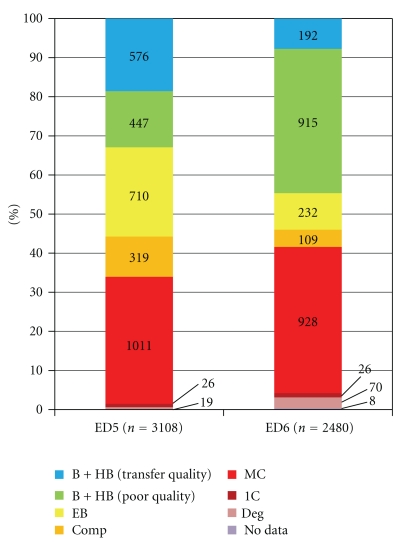
Comparison of embryo morphology on ED5 and ED6. Morphological categories on ED5 and ED6 are shown by percentage in stacked columns. Numbers indicate the number of embryos in each category. HB = hatching blastocyst, B = blastocysts, EB = early blastocyst, comp = compacted, MC = multicell, 1C = one cell, deg = degenerated.

Having established that nonviable ED6 embryos retain the capacity to give rise to hESC lines, albeit at low frequency, we carried out a retrospective chart analysis of the morphological categories in embryos generated for clinical use. Our goal was to increase the efficiency of hESC derivation by the earlier identification (ED5) of embryos that do not improve further but still have the capacity to give rise to hESC lines. Embryo scoring is used as a prognostic tool in IVF treatment; here we compared embryo scores at ED5 with scores at ED6 to assess morphological criteria that would be indicative of nonviability on ED5. We have analyzed and compared morphological categories from ED5 and ED6 as cohort populations since embryos were scored in group culture rather than individually. From a starting number of 3554 embryos, 446 were transferred or cryopreserved on ED3 and were not part of further analysis. Of 3108 embryos evaluated on ED5, 628 were transferred or cryopreserved (all good quality embryos meeting criteria for transfer) (Figure 4). The remaining 2480 embryos were deemed unsuitable for clinical use on ED5 and were reevaluated on ED6. Upon reevaluation on ED6, 192 transfer-quality embryos were cryopreserved while the remaining 2288 embryos were discarded as being unsuitable for clinical use. Of these, 915 were graded as blastocysts or hatching blastocysts not of transfer quality and were thus given grades for overall quality and ICM/TE quality (Table 1). Most of these embryos were classified as poor (898/915; >99%) and only 17/915 (less than 0.02%) were graded as good or fair quality on ED6. Therefore, embryo quality at ED5 is highly correlated with embryo quality at ED6 indicating that criteria for nonviability could be established at ED5.

## 4. Discussion

 In this study, we derived two new hESC lines from ED6 nonviable embryos with the overall derivation efficiency of 1.25% (2/159) for compacted and/or cavitated embryos. This is in line with derivation efficiencies previously reported for nonviable, organismically dead or poor quality embryos: 0.75–2.66%. Not surprisingly, these derivation efficiencies are somewhat lower than derivation success rates from viable blastocysts (6–50% depending on culture conditions) [27, 28]. 

We have demonstrated that CU1 and CU2 lines show properties of hESC and differentiate into derivatives of all three embryonic germ layers *in vitro*. However, differentiation *in vivo* failed as no teratomas were isolated. This is in contrast with previous reports of hESC lines derived from nonviable embryos that were shown to give rise to differentiated tissues *in vivo* [23, 26] but in agreement with several other studies that have reported lack of teratoma formation or lack of* in vivo *differentiation for lines derived from embryos of low quality [29–31]. In our case, the failure of teratoma formation may be due to technical limitations or could represent a restriction in developmental potential, although the *in vitro* differentiation studies would argue against the latter explanation. Additional investigation will be required in order to determine whether failed *in vivo* differentiation or a lower success of teratoma formation is a property of hESC derived from nonviable embryos. Nevertheless, hESC lines derived from arrested nonviable embryos, including those reported here show characteristics and *in vitro* differentiation potential comparable to hESC derived from surplus viable embryos [23, 26].

Abnormal karyotypes have been reported in hESC after extended periods of culture, frequently involving chromosomes 12, 17, and X as well as other chromosomes, including chromosome 18, less frequently [32, 33]. These chromosomal anomalies are thought to be the result of adaptive changes that occur with long-term culture. The low level of mosaicism for monosomy 18 observed in CU2 could have arisen during culture, but because it was observed in cells at an early pass number, it could also have been present in the embryo used to derive the cell line. Although a high level of aneuploidy might be expected in arrested or abnormal embryos, this has generally not been the case in hESC derived from them (see [26]; this study), indicating that there are embryos with normal karyotypes, or at least embryos containing some cells with normal karyotypes, among poor quality embryos.

By the derivation of CU1, we have demonstrated that poor quality DC blastocysts on ED6 can retain their capacity to give rise to hESC (Figure 1(a)). The CU2 line originated from a poor quality ED6 embryo with a small cavity (Figure 1(b)) which was not given grades for the ICM/TE quality. From our previous study, we can estimate that poor quality cavitated embryos contain between 5 and 64 living cells at ED6 [20]. In our retrospective analysis, we have focused on further defining criteria of developmental arrest for ED5 embryos to improve derivation efficiency from nonviable embryos. Our analysis shows that only a small proportion of embryos deemed unsuitable for embryo transfer at ED5 improve to transferable quality by ED6 (192/2480; 7.7%). This suggests that the majority of embryos could be considered nonviable and that if embryos were individually cultured, a minimum clinical grade could be identified on ED5 that would correlate with lack of viability on ED6. By targeting derivation efforts on a subgroup of nonviable embryos that have no potential to improve with extended *in vitro* culture and yet could yield hESC lines, success rates could be increased. 

We have observed that published criteria for deeming embryos poor quality are quite variable [31, 34–37] as several of these reports lacked sufficient information to conclude whether embryos used for derivation had an irreversible loss of integrated organismic function. Two studies report on hESC derivation from discarded day 3 embryos with low morphological scores [34] or poor quality day 3 embryos [36] that develop into blastocysts after 48 hours culture. Recently, a universal minimum information (MI) convention for reporting on hESC derivation was proposed [38, 39] and used in a study to classify poor quality blastocysts that subsequently yielded 17 hESC lines [37]. However, it remains unclear which of the 17 blastocysts were from cleavage embryos cultured for 48 hours versus thawed blastocysts and thus from arrested versus surplus embryos. While information on the quality of an embryo used to establish hESC lines should be readily available, it would be practical if the blastocyst grading system proposed in this MI convention included overall embryo quality scores such as those employed in IVF clinics. Information on quality of embryos used for hESC derivation would be pertinent to eventual therapeutical application in that patients could be fully informed as to the source of cells used in their therapy. 

In summary, we derived and characterized two new hESC lines from discarded, nonviable ED6 embryos. Our retrospective study suggests that the grading system currently in use could be employed for the early identification of irreversibly arrested embryos suitable for hESC derivation at ED5. Embryos with such characteristics could be used for large-scale derivation of hESC without the destruction of vital human embryos.

## 5. Conclusions

Previously, we proposed that the ethical criteria applied to essential organ donation could be extended to derivation of hESC lines from embryos with an irreversible loss of integrated organismic function produced during routine *in vitro *fertilization. This study shows the feasibility of deriving hESC lines from such embryos using standard grading systems for identification of suitable embryos. Ethical considerations and the current legislative climate make alternative sources for deriving genetically diverse hESC lines for research and therapeutic purposes a priority.

## Supplementary Material

Figure S1. Karyotypes of CU1 (passage 9) (A) and CU2 (passage 8) (B). Verbatim lab reports.
Figure S2. Characterization of CHB1 (A-J) and CU2 (K-O) hESC lines. Immunohistochemical analysis with DAPI counterstain revealed that hESC colonies expressed pluripotency markers POU5F1/OCT4 (A,K), SSEA-4 (B,L), TRA1-60 (D), TRA-1-81 (E) (green fluorescence) and were negative for differentiation marker SSEA-1 (C). hESC cultured as embryoid bodies differentiated into all three germ layers: hematoxylin and eosin (F) and Masson's trichrome (G), immunohistochemistry for neuron specific beta-III tubulin (TUJ-1) (H,M), alpha fetoprotein (AFP) (I,N) and smooth muscle actin (SMA) (J,O) (green fluorescence). Scale bar-100 *μ*m.

## Figures and Tables

**Table 1:** Quality scores of 915 embryos graded B (blastocysts) or HB (hatching blastocysts) on ED6 and deemed unsuitable for clinical use.

	ED6 ICM/TE score^a^										
ED6 grade	AB	BB	BC	BD	CB	CC	CD	DB	DC	DD	Total
Good	1										1
Fair		5	1		2	4		1	1	1	16
Poor			1	2	3	223	113	20	76	460	898
Total	1	5	2	2	5	227	113	21	77	461	915

^a^The first letter represents the quality of the ICM and the second letter the quality of TE.
