# Supplementary material for: Derivation of Two New Human Embryonic Stem Cell Lines from Nonviable Human Embryos
Source: Stem Cells Int. 2011 May 22;2011:765378. doi: 10.4061/2011/765378 (PMC3118293; doi:10.4061/2011/765378)
Supplement: Supplementary file 1 — Figure S1. Karyotypes of CU1 (passage 9) (A) and CU2 (passage 8) (B). Verbatim lab reports. Figure S2. Characterization of CHB1 (A-J) and CU2 (K-O) hESC lines. Immunohistochemical analysis with DAPI counterstain revealed that hESC colonies expressed pluripotency markers POU5F1/OCT4 (A,K), SSEA-4 (B,L), TRA1-60 (D), TRA-1-81 (E) (green fluorescence) and were negative for differentiation marker SSEA-1 (C). hESC cultured as embryoid bodies differentiated into all three germ layers: hematoxylin and eosin (F) and Masson's trichrome (G), immunohistochemistry for neuron specific beta-III tubulin (TUJ-1) (H,M), alpha fetoprotein (AFP) (I,N) and smooth muscle actin (SMA) (J,O) (green fluorescence). Scale bar-100 μm. [file 765378.f1.pdf]

Figure S1

A

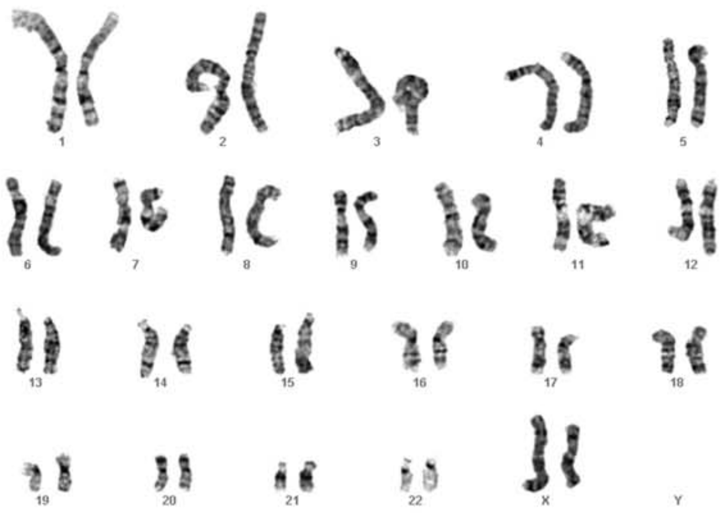

CU1 p9
Results: 46,XX
Interpretation: Cytogenetic analysis of cultured embryonic stem cells showed a normal female karyotype in all cells analyzed.

B

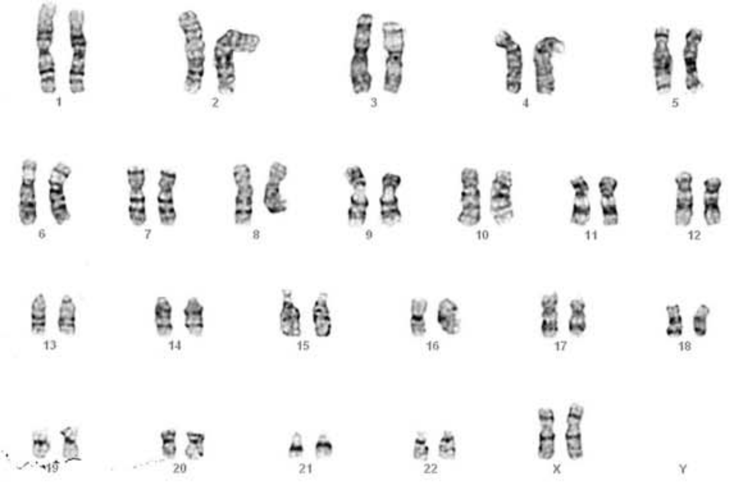

CU2 p8
Results: 46,XX
Interpretation: Cytogenetic analysis of cultured embryonic stem cells showed a NORMAL female karyotype in the majority of the cells analyzed. Three cells showed a recurrent loss of chromosome 18. This was observed in the first 20 cells analyzed. Additional analysis of 11 more cells showed a normal female karyotype. The loss of chromosome 18 is more likely attributed to technical preparation of the slides. However, low level of mosaicism cannot be ruled out.
