# Supplementary figures and images for: Derivation of Two New Human Embryonic Stem Cell Lines from Nonviable Human Embryos
Source: Stem Cells Int. 2011 May 22;2011:765378. doi: 10.4061/2011/765378 (PMC3118293; doi:10.4061/2011/765378)

**Figure S2**

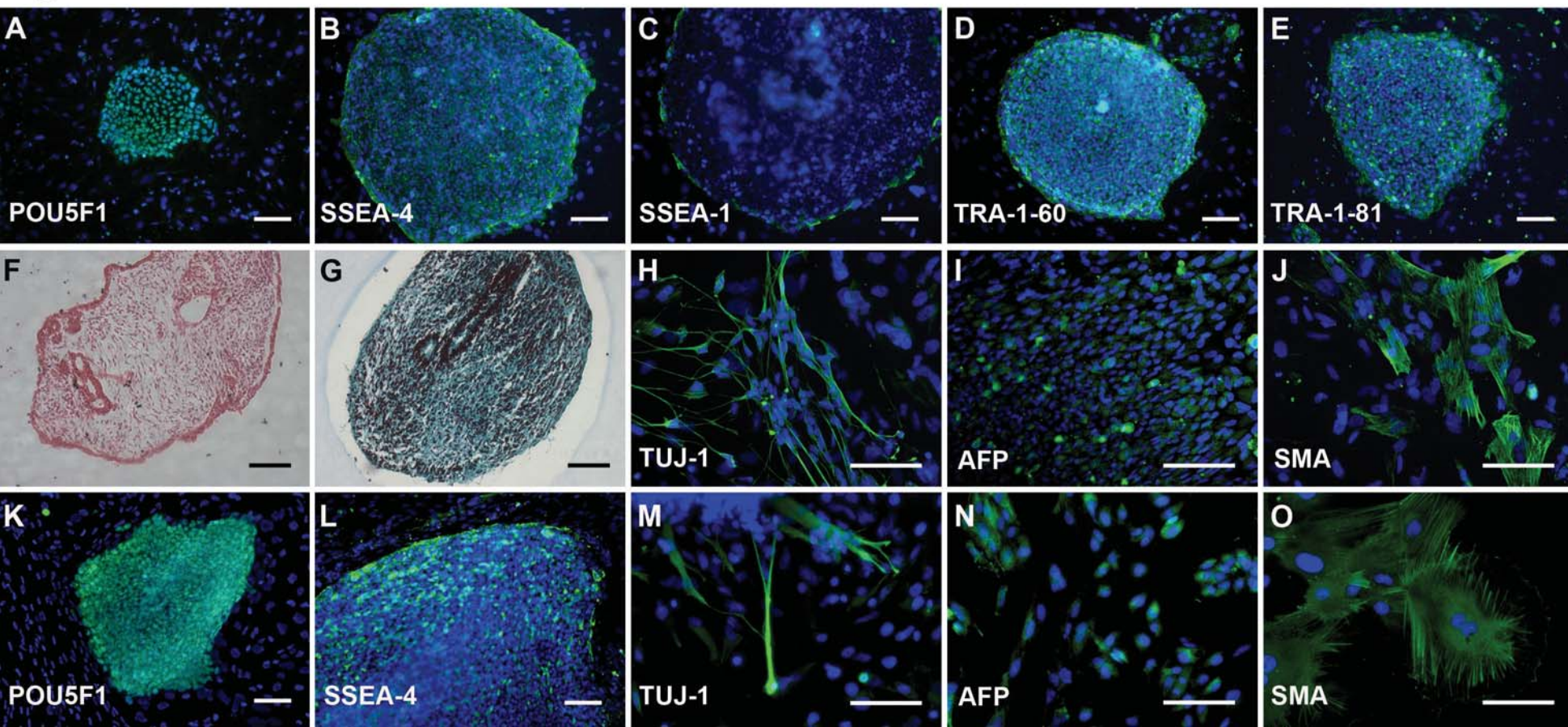

Supplement: Supplementary file 2 [file 765378.f2.pdf]
